# Molecular Characterization of *Mycobacterium tuberculosis* Isolates From West of Iran Using Mycobacterial Interspersed Repetitive Unit‐Variable Number Tandem Repeats: A Cross‐Sectional Study

**DOI:** 10.1002/hsr2.72473

**Published:** 2026-05-13

**Authors:** Mohammad Yazdanmanesh, Keyvan Tadayon, Hossein Kazemian, Nilufar Sadouqi, Nader Mosavari

**Affiliations:** ^1^ Bovine Tuberculosis Reference Laboratory, Agricultural Research, Education and Extension Organization (AREEO) Razi Vaccine and Serum Research Institute Karaj Iran; ^2^ Clinical Microbiology Research Center Ilam University of Medical Sciences Ilam Iran

**Keywords:** MIRU‐VNTR, *Mycobacterium tuberculosis*, rifampicin

## Abstract

**Background and Aims:**

Tuberculosis (TB) remains a major global health problem and poses significant challenges for diagnosis and treatment. The World Health Organization (WHO) recommends the use of molecular methods for the diagnosis and genotyping of *Mycobacterium tuberculosis* complex (MTBC). Mycobacterial interspersed repetitive unit‐variable number tandem repeats (MIRU‐VNTR) has proven to be a valuable method for studying the genetic diversity of *Mycobacterium tuberculosis* (MTB). To the best of our knowledge, no previous study has investigated MTB diversity in Ilam, west of Iran. Therefore, this study aimed to investigate the genetic diversity of *Mycobacterium tuberculosis* isolates and the occurrence of rifampicin‐resistant *Mycobacterium tuberculosis* (RR‐TB).

**Methods:**

In total, 643 suspected cases of TB were collected from March 2022 to November 2023. Acid‐fast bacilli (AFB) were detected using Ziehl–Neelsen (ZN) staining, fluorochrome staining, and culture on Löwenstein–Jensen medium. GeneXpert MTB/RIF method was applied to detect MTB and RR‐TB. Finally, 15‐locus‐based MIRU‐VNTR was used for molecular genotyping.

**Results:**

Out of 643 specimens, 20/643 (3.11%) specimens were diagnosed as positive for MTB by culture. GeneXpert MTB/RIF confirmed RR‐TB in one sample. Positive isolates were placed in nine different clusters using MIRU‐VNTR genotyping. All isolates were not assignable to the sublineages in the MIRU‐VNTRplus database but were close to the Delhi/CAS.

**Conclusion:**

The high clustering rate in our results indicates that TB transmission in this region originates from multiple sources and reflects active transmission rather than reactivation of latent TB. In addition, the lineages of MTB isolates identified in Ilam were closely related to those reported from other Iranian provinces and neighboring countries, indicating epidemiological links. Improving diagnostic capacity and implementing effective control measures are essential to reduce TB transmission.

## 1 Introduction

*Mycobacterium tuberculosis* (MTB) is the main cause of tuberculosis (TB). TB can affect individuals of all ages, and it has the potential to harm nearly all human organs, particularly the lungs [1, 2]. Pulmonary TB is the most frequent and clinically significant form of TB, therefore, it should be distinguished from other types of TB due to its highest incidence and public health importance [3]. Drug‐resistant tuberculosis (DR‐TB) continues to be a source of concern in TB treatment and disease management [4]. Rifampicin (RIF) is one of the most important anti‐TB drugs used in the management of TB [5]. Almost 90% of rifampicin‐resistant *Mycobacterium tuberculosis* (RR‐TB) strains are also resistant to isoniazid, thus, RR‐TB has been considered as a surrogate marker for drug‐resistant and multidrug‐resistant TB (MDR‐TB) [6, 7]. TB is responsible for approximately three deaths every minute worldwide. According to the global TB Report 2022 by the World Health Organization (WHO), there were 10.6 million new TB cases, 410,000 MDR/RR‐TB, and 1.6 million TB deaths [8, 9]. More than 90% of new TB cases and deaths occur in low‐/middle‐income countries. Poverty and poor living conditions can weaken the immune system and raise the risk of TB [1, 10]. According to the Global TB Report 2024, the prevalence of TB in Iran was estimated to be 12/100,000 people [11]. WHO recommends the GeneXpert MTB/RIF assay as one of the best molecular methods for the rapid diagnosis of RR/MDR‐TB [12, 13]. Molecular genotyping of MTBC is a valuable adjunct in TB control for tracking disease transmission [14]. Currently, several typing methods are used for MTBC, among which *IS6110*‐restriction fragment length polymorphism, spoligotyping, and mycobacterial interspersed repetitive unit‐variable number tandem repeat (MIRU‐VNTR) are the most widely used. Among these methods, MIRU‐VNTR genotyping is one of the most widely used techniques [15]. The advantages of this method include fast performance, easy analysis, reliable information on the phylogeographic distribution of MTB strains, and standard criteria for the identification of the different MTB lineages and sublineages [15, 16]. Ilam Province is located in western Iran and borders two regions with a relatively high TB burden (Kermanshah and Iraq). Moreover, millions of people travel through this province due to annual religious ceremonies such as Arbaeen pilgrimage. To our knowledge, no study has investigated MTB diversity in this province. Therefore, to gain insight into the molecular epidemiology of MTB, 15 loci MIRU‐VNTR was performed in this study. GeneXpert MTB/RIF was also used to identify RR‐TB.

## 2 Materials and Methods

### 2.1 Sample Collection and Processing

In total, 643 clinical samples were collected from March 2022 to November 2023 from referred to the Ilam TB diagnostic laboratory. According to national TB diagnostic guidelines, patients with clinical symptoms such as persistent cough (2 weeks or more), chest pain, fever, night sweats, weight loss, hemoptysis, or abnormal chest X‐ray findings are considered suspected cases of TB [17]. For sampling, three sputum specimens were obtained from each suspected TB patient in sterile bottles. For reference standard testing, all clinical specimens were first digested with *N*‐acetyl‐L‐cysteine NaOH method (final NaOH concentration: 1%). After decontamination, all samples were stained using Ziehl–Neelsen (ZN) and fluorochrome to detect Acid‐fast bacilli (AFB). Furthermore, sediments of all samples were inoculated on Löwenstein–Jensen (LJ) medium for the detection of MTB, and incubated at 37°C for 8 weeks and checked for growth weekly. All laboratory procedures were performed in a biosafety level 3 (BSL‐3) facility at the Razi Vaccine and Serum Research Institute.

### 2.2 GeneXpert MTB/RIF Assay

Positive samples were analyzed by GeneXpert MTB/RIF assay (Cepheid, CA, USA). The GeneXpert MTB/RIF assay was performed according to the manufacturer's instructions. Briefly, 2 mL of GeneXpert MTB/RIF sample reagent buffer was mixed with 1 mL of sputum specimen using a sterile pipette and shaken 10–20 times and allowed to incubate at room temperature for 10 min, following which it was shaken again, and allowed to stand at room temperature for 5 min. Then, 2 mL of the mixture was transferred to the test cartridge and placed into the GeneXpert device. Lastly, the results were generated and created after 2 h [18].

### 2.3 Molecular Identification

#### 2.3.1 DNA Extraction

Genomic DNA was extracted from colonies using the cetyltrimethylammonium bromide (CTAB) method as previously described [19]. Briefly, two to three loopfuls of the MTB culture were suspended in Tris EDTA buffer (10 mM Tris‐HCl [pH 8.0], 1 mM EDTA) and placed at 80°C for 20 min to kill the bacteria. DNA was extracted from the heat‐killed MTB suspension using an enzymatic method involving lysozyme, and the DNA was then purified with CTAB‐NaCl and chloroform‐isoamyl alcohol. NanoDrop spectrophotometry (ND‐1000 WOC, Thermo Fisher Scientific, United States) was used to measure the quality of genomic DNA.

#### 2.3.2 Molecular Genotyping

MIRU‐VNTR genotyping was performed using the standardized 15‐locus protocol as previously described [20]. Primers specific to the flanking regions of the VNTR loci were used as described by Supply et al. [20], and were commercially synthesized (SinaClon, Tehran, Iran). Each polymerase chain reaction (PCR) reaction contained 8 μL PCR Master Mix (SinaClon, Tehran, Iran), 1 μL of each forward and reverse primer (10 pmol/μL), 3 μL of the DNA template (20 ng/μL), 0.6 μL dimethyl sulfoxide, and finally 2.4 μL distilled water suitable for molecular tests. PCR amplification was performed in a Thermal Cycler (Eppendorf PRO S 6325 Thermal, Germany) under the following conditions: 95°C for 5 min, 35 cycles of 95°C for 30 s, 64°C for 30 s, 72°C for 30 s, and 72°C for 10 min. Amplicon sizes were confirmed by electrophoresis on a 1% agarose gel and visualized under UV light. The MIRU‐VNTRplus software was used to assess genetic relationships among all isolates, and ultimately, a dendrogram was constructed following the instructions provided by MIRU‐VNTRplus (available at https://www.miru-vntrplus.org/MIRU/index.faces). The software default suggested a discrimination cut‐off of 0.17 for strains. The allelic diversity was evaluated using the Hunter–Gaston discriminatory index (HGDI) (available at http://insilico.ehu.es/mini_tools/discriminatory_power/?show=formula). Based on this index, the loci were designated as highly (HGDI > 0.6), moderately (HGDI 0.6–0.3), and poorly discriminatory (HGDI < 0.3) as described previously [21]. Ultimately, the recent transmission rate was calculated using the formula: (TC − NC)/TA, where TC is the total number of clustered isolates, NC is the number of clusters, and TA is the total number of analyzed isolates [22]. This formula provides a descriptive approximation of the proportion of the recent transmission rate.

### 2.4 Statistical Analysis

Data were analyzed descriptively using Microsoft Excel (version 2021), and frequencies, percentages, means, and standard deviations (mean ± SD) were calculated. No inferential statistical tests were performed, as the study design was descriptive in nature. Genetic analysis of MIRU‐VNTR data, including clustering, minimum spanning tree construction, and calculation of HGDI, was performed using the MIRU‐VNTRplus online analysis tool (https://www.miru-vntrplus.org). The reporting of descriptive statistics followed the Statistical Analyses and Methods in the Published Literature (SAMPL) guidelines for transparent presentation of data.

## 3 Results

**Figure 1:**
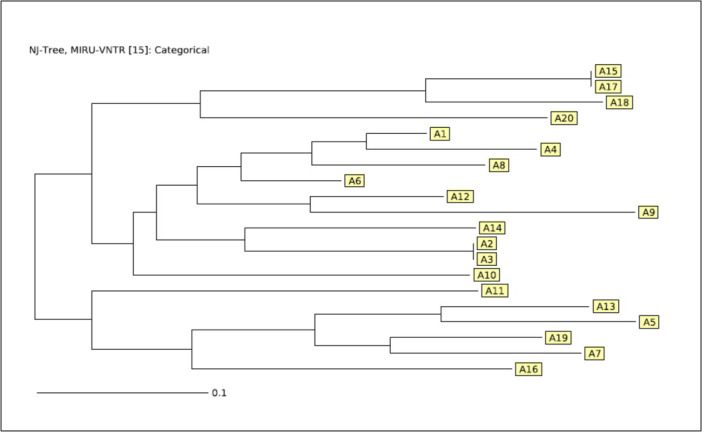
Genetic relatedness of 20 MTB isolates by MIRU‐VNTR genotyping (0.17 cut‐off). A dendrogram was created to display clustering of isolates (A1–A20). Nine distinct clusters were identified.

**Figure 2:**
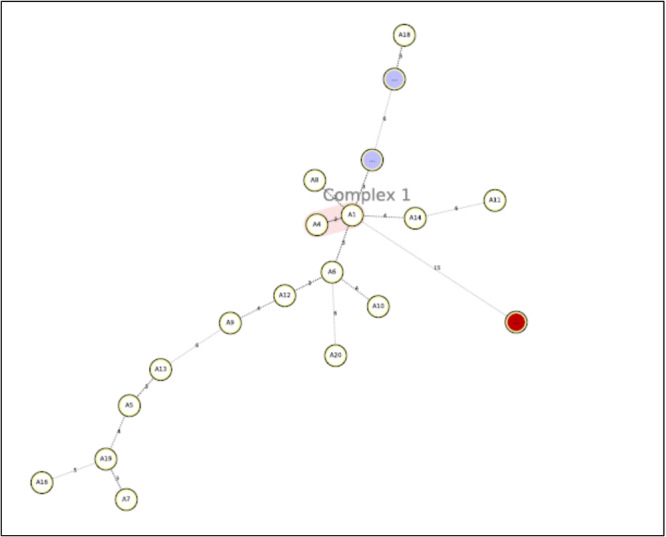
Minimum spanning tree of MTB isolates based on MIRU‐VNTR profiles. Each circle represents one genotype, and the presence of a single clonal complex indicates high genetic similarity among isolates.

Among the 643 clinical specimens, 19 (2.95%) were AFB positive by smear microscopy. The comparison of MTB detection methods is presented in Table 1. Culture on LJ medium identified 20 (3.11%) MTB‐positive isolates. All culture‐positive isolates were further confirmed using the GeneXpert MTB/RIF assay, which detected rifampicin resistance in one isolate. Subsequently, all 20 culture‐confirmed isolates were subjected to MIRU‐VNTR genotyping for molecular characterization. The positive patients had a mean age of 49.3 ± 11.9 years (range 29–78), and 15/20 patients were male (75%) and 5/20 were female (25%). The summary of demographic characteristics of MTB‐positive patients is presented in Supporting Information S1: Table A, while detailed individual‐level data (age and sex for each patient) are provided in Supporting Information S1: Table B. Based on the MIRU‐VNTR genotyping results, all 20 culture‐positive MTB isolates were categorized into 9 different clusters (Figure 1). The detailed distribution of clusters and the proportion of clustered isolates are summarized in Supporting Information S1: Table C. The largest clusters were 2 clusters each consisting of 4/20 isolates, while there were also 2 clusters containing 3/20 isolates. Additionally, there was 1 cluster containing 2/20 isolates and 4 clusters with 1/20 isolates. Furthermore, discriminatory power of MIRU‐VNTR was high (HGDI: 0.8954). In addition, transmission rate estimation showed a high rate of recent transmission (55%, 11/20 cases). As shown in the minimum spanning tree (Figure 2), there was one clonal complex, indicating high clonality.

**Table 1:** Comparison of MTB detection from clinical specimens using AFB smear microscopy, culture, and GeneXpert method.

Case	AFB smear	Culture	GeneXpert
A1	+	+	+
A2	+	+	+
A3	+	+	+
A4	+	+	+
A5	+	+	+
A6	+	+	+
A7	+	+	+
A8	+	+	+
A9	+	+	+
A10	+	+	+
A11	+	+	+
A12	+	+	+
A13	+	+	+
A14	+	+	+
A15	+	+	+
A16	−	+	+
A17	+	+	+
A18	+	+	+
A19	+	+	+
A20	+	+	+

## 4 Discussion

**Figure 3:**
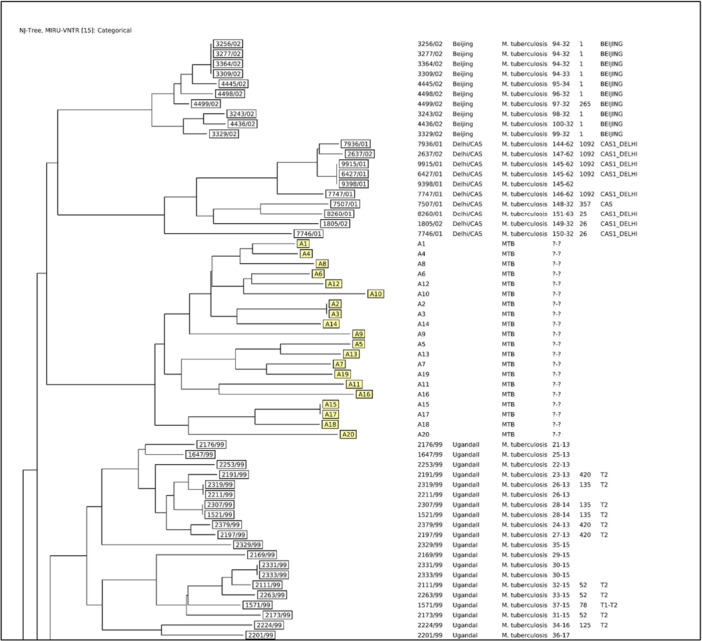
Genetic relationships between the study isolates (A1–A20) and previously studied isolates presented in MIRU‐VNTRplus website. The Ilam isolates show close similarity to the Delhi/CAS lineage.

The aim of TB control programs is to eradicate the disease by interrupting the transmission chain, which can be effectively achieved through rapid diagnosis and conclusive treatment of active TB cases [23]. The present study appears to be the first study reporting MIRU‐VNTR profiles of MTB in Ilam province. The results of 20 MTB isolates showed 9 clusters. The largest and smallest MIRU‐VNTR clusters contained 4 and 1 isolates, respectively. Many studies have demonstrated the effect of MTB genetic diversity on virulence factors [24]. This high clustering rate observed in Ilam Province could be attributed to Arbaeen pilgrimage and border with Iraq. In recent years, urbanization, the border with Iraq, and the Arbaeen pilgrimage have made Ilam province one of the busiest provinces in Iran. Therefore, this province receives a large number of international travelers. Consequently, migration may influence TB transmission patterns and epidemiological characteristics in this province. In recent decades, molecular genotyping of MTB and RR/MDR‐TB has been carried out in different areas of Iran, such as Hormozgan, a province with a highly active port for trading, fishing, oil industry, and tourism [25]. Kermanshah, a province border with Iraq [26], Alborz, a multiethnic region province with the large number of Afghan settlers and high TB burden [27], Isfahan, a tourist city [28], and Tehran capital of Iran, is a multiethnic region with its population consisting of almost all the country's ethnicities and foreign nationals dominated by Afghan and Iraqi settlers [22], but there is no information regarding Ilam Province. Therefore, the current study was carried out in order to assess the situation of TB in this province. In our study, the MIRU‐VNTR profiles were not assignable to the sublineages in the MIRU‐VNTRplus database, but all isolates were close to Delhi/CAS (Figure 3). This may be due to the small sample size in our study. In a study by Bakhtiyariniya and colleagues, from Khuzestan (a province in the south of Ilam and borders with Iraq) 34.5% of isolates were Delhi/CAS [29]. Another study conducted by Riyahi Zaniani and colleagues from Isfahan (a tourist city in the center of Iran) found that 28.57% of isolates were Delhi/CAS [28]. Haeili and colleagues indicate that Delhi/CAS was more frequent (45%) in Sistan‐Baluchestan (South‐East of Iran which shares borders with Afghanistan and Pakistan, two high‐burden TB regions) [30]. All in all, in Iran, the lineages of New‐1 and Delhi/CAS are more adapted to the Iranian population [30, 31, 32]. According to our data, one case (A16) was RR‐TB, exhibiting a unique pattern in our MIRU‐VNTR genotyping (Figure 2). This case was smear negative, which may be attributed to latent TB or unsuccessful treatment outcome.

In conclusion, 15‐locus MIRU‐VNTR genotyping is a straightforward, dependable, and consistent technique that holds significant potential for genetic analysis and monitoring epidemiological occurrences like transmission dynamics. This is the first study to reveal the diversity of MTB strains in Ilam province. The high rate of clustering in our results shows that TB transmission in Ilam is mostly related to the newly transmitted disease, and reactivation of previous infection plays a minor role. In this region, due to the lack of equipment and adequately of equipped medical centers, timely detection and treatment of TB have faced a problem. Further studies are required to provide high‐resolution insights into the population structure of TB in this province. Furthermore, MDR‐TB remains a continuously evolving health problem. Efficient diagnostic procedures such as GeneXpert may be highly valuable in detecting RR/MDR‐TB cases, which can help manage the infection.

## Author Contributions

**Mohammad Yazdanmanesh:** writing – original draft, investigation. **Keyvan Tadayon:** supervision, project administration. **Hossein Kazemian:** investigation, methodology. **Nilufar Sadouqi:** writing – original draft, validation, software. **Nader Mosavari:** writing – review and editing, project administration.

## Funding

The authors have nothing to report.

## Disclosure

The lead authors Keyvan Tadayon and Nader Mosavari affirm that this manuscript is an honest, accurate, and transparent account of the study being reported; that no important aspects of the study have been omitted; and that any discrepancies from the study as planned (and, if relevant, registered) have been explained.

## Ethics Statement

This study was approved by the Ethics Committee of Razi Vaccine and Serum Research Institute (IR.RVSRI.REC.1402.001).

## Consent

Written informed consent was obtained from all individual participants included in the study.

## Conflicts of Interest

The authors declare no conflicts of interest.

## Supporting information


**Table S1:** Demographic characteristics of MTB‐positive patients (n = 20).
**Table S2:** Detailed distribution of MTB‐positive patients (age and sex).
**Table S3:** Distribution of MTB isolates by MIRU‐VNTR genotyping and clustering analysis. The table presents the number and percentage of isolates in each cluster, the total proportion of clustered isolates, and the estimated recent transmission rate.

## Data Availability

Data supporting this study's findings are available upon reasonable request from the corresponding authors. Nader Mosavari and Keyvan Tadayon had full access to all of the data in this study and take complete responsibility for the integrity of the data and the accuracy of the data analysis.
