# Supplementary material for: Molecular Characterization of Mycobacterium tuberculosis Isolates From West of Iran Using Mycobacterial Interspersed Repetitive Unit‐Variable Number Tandem Repeats: A Cross‐Sectional Study
Source: Health Sci Rep. 2026 May 13;9(5):e72473. doi: 10.1002/hsr2.72473 (PMC13172268; doi:10.1002/hsr2.72473)
Supplement: Supplementary file 1 — Table S1: Demographic characteristics of MTB‐positive patients (n = 20). Table S2: Detailed distribution of MTB‐positive patients (age and sex). Table S3: Distribution of MTB isolates by MIRU‐VNTR genotyping and clustering analysis. The table presents the number and percentage of isolates in each cluster, the total proportion of clustered isolates, and the estimated recent transmission rate. [file HSR2-9-e72473-s001.docx]

Supplementary Table A. Demographic characteristics of MTB-positive patients (n = 20).

| **Variable** | **Value** |
| --- | --- |
| Age, mean ± SD | 49.3 ± 11.9 years |
| Age, median (range) | 49 (29–78) years |
| Sex, n (%) | Male: 15 (75%), Female: 5 (25%) |

Supplementary Table B. Detailed distribution of MTB-positive patients (age and sex).

| **Patient ID** | **Age** | **Sex** |
| --- | --- | --- |
| A1 | 57 | Male |
| A2 | 49 | Male |
| A3 | 64 | Male |
| A4 | 37 | Male |
| A5 | 70 | Male |
| A6 | 35 | Male |
| A7 | 53 | Male |
| A8 | 58 | Male |
| A9 | 49 | Male |
| A10 | 78 | Male |
| A11 | 50 | Male |
| A12 | 49 | Male |
| A13 | 47 | Male |
| A14 | 39 | Male |
| A15 | 45 | Male |
| A16 | 29 | Female |
| A17 | 39 | Female |
| A18 | 40 | Female |
| A19 | 49 | Female |
| A20 | 48 | Female |

Supplementary Table C. Distribution of MTB isolates by MIRU-VNTR genotyping and clustering analysis. The table presents the number and percentage of isolates in each cluster, the total proportion of clustered isolates, and the estimated recent transmission rate.

| **Cluster** | **No. of isolates** | **% of total (n=20)** |
| --- | --- | --- |
| Cluster 1 | 4 | 20% |
| Cluster 2 | 4 | 20% |
| Cluster 3 | 3 | 15% |
| Cluster 4 | 3 | 15% |
| Cluster 5 | 2 | 10% |
| Cluster 6–9 | 1 each | 20% |
| **Total clustered isolates** | 16 | 80% |
| **Transmission rate** | 55% (11/20) |  |
